# Cognitive biases encountered by physicians in the emergency room

**DOI:** 10.1186/s12873-022-00708-3

**Published:** 2022-08-26

**Authors:** Kotaro Kunitomo, Taku Harada, Takashi Watari

**Affiliations:** 1 Department of General Medicine, Kumamoto Medical Center, Kumamoto, Japan; 2 Department of General Medicine, Koto Toyosu Hospital, Tokyo, Japan; 3 General Medicine Center, Shimane University, 89-1, Enya-cho, Izumo shi, Shimane 693-8501 Japan; 4 Department of Medicine, University of Michigan Medical School, Ann Arbor, MI USA

**Keywords:** Diagnostic error, Medical error, Cognitive biases, Medical safety

## Abstract

**Background:**

Diagnostic errors constitute an important medical safety problem that needs improvement, and their frequency and severity are high in emergency room settings. Previous studies have suggested that diagnostic errors occur in 0.6-12% of first-time patients in the emergency room and that one or more cognitive factors are involved in 96% of these cases. This study aimed to identify the types of cognitive biases experienced by physicians in emergency rooms in Japan.

**Methods:**

We conducted a questionnaire survey using Nikkei Medical Online (Internet) from January 21 to January 31, 2019. Of the 159,519 physicians registered with Nikkei Medical Online when the survey was administered, those who volunteered their most memorable diagnostic error cases in the emergency room participated in the study. EZR was used for the statistical analyses.

**Results:**

A total of 387 physicians were included. The most common cognitive biases were overconfidence (22.5%), confirmation (21.2%), availability (12.4%), and anchoring (11.4%). Of the error cases, the top five most common initial diagnoses were upper gastrointestinal disease (22.7%), trauma (14.7%), cardiovascular disease (10.9%), respiratory disease (7.5%), and primary headache (6.5%). The corresponding final diagnoses for these errors were intestinal obstruction or peritonitis (27.3%), overlooked traumas (47.4%), other cardiovascular diseases (66.7%), cardiovascular disease (41.4%), and stroke (80%), respectively.

**Conclusions:**

A comparison of the initial and final diagnoses of cases with diagnostic errors shows that there were more cases with diagnostic errors caused by overlooking another disease in the same organ or a disease in a closely related organ.

**Supplementary Information:**

The online version contains supplementary material available at 10.1186/s12873-022-00708-3.

## Background

Diagnostic errors are among the most important medical safety problems that need to be addressed, and the emergency room is one of the most common places where their frequency and severity are high [1]. This is because the emergency room is a workplace with frequent interruptions and one that requires multitasking; moreover, it is necessary to make decisions quickly for first-time emergency patients who have not been diagnosed [2–6]. According to dual process theory, we conducted clinical decision making in both System 1, which is intuitive, heuristic, and unconscious, and System 2, which is analytical and logical conscious thinking [7]. The factor that predisposes people to think in a way that causes them to fail in their decision making is called cognitive bias [8]. In the emergency room, patients are often diagnosed using System 1, which is affected by cognitive biases [9, 10]. Despite that cognitive biases are common and unrelated to knowledge, most people are not easily aware of their own cognitive biases. In fact, previous studies have suggested that diagnostic errors occur in 0.6-12% of the patients in the emergency room [11, 12] and that one or more cognitive factors are related in 96% of these cases [13].

In Japan, it has been noted that efforts to improve diagnostic errors have been slow due to the influence of multiple factors, including medical education, the healthcare system, and a culture of shame [14]. In addition, although there is a recent report from Japan on the analysis of diagnostic errors by residents [15], few studies have addressed diagnostic errors in Japan. In addition, regarding emergency rooms, single-center studies have quantified the cognitive biases that are likely to occur [16], but there are no quantified studies set in Japan.

Therefore, identifying cognitive biases in the emergency room is important for reducing errors. This study aimed to identify the types of cognitive biases experienced by physicians in emergency rooms in Japan.

## Methods

### Study design

We performed a questionnaire survey using Nikkei Medical Online (Internet), a free membership portal of medical information for medical doctors/medical workers, from January 21 to January 31, 2019. In undertaking this work, we followed the Declaration of Helsinki and the guidelines of strengthening the reporting of observational studies in epidemiology (STROBE).

### Study sample

Of the 159,519 physicians registered with Nikkei Medical Online when the survey was administered, physicians who volunteered their most memorable cases of diagnostic errors in the emergency room were chosen.

### Data collection

The survey categories included physicians’ background (age, number of years since graduation, number of years of post-graduate experience at the time of the encounter, and physicians’ specialty), healthcare provider environment (days of the week, time of day, clinical environment, day and night shifts, and size of the healthcare facility), and factors related to diagnostic errors (type of error, location of error, frequency of error, initial diagnosis, final diagnosis, time for detection, environmental factors, information collecting factors, and information integrating factors).

The inclusion criterion were that the respondents were physicians and that the location where the diagnostic error occurred was the emergency room. We considered as diagnostic errors those responses that corresponded to either Missed Diagnosis, Wrong Diagnosis, or Delayed Diagnosis [16]. Exclusion criteria were that the error did not occur in the emergency room, important data were incomplete (incorrect entry, missing data), and the setting was a clinic. The types of cognitive bias presented in the questionnaire and their definitions are explained in Table 1. The list of representative cognitive biases distributed to participants.

**Table 1:** Types of cognitive bias presented in the questionnaire

Cognitive biases	Explanation
Availability bias	The tendency to instinctively think of things that come to mind easily as being more representative than they actually are.
Overconfidence bias	The tendency to have an inaccurate and false opinion about one’s self
Anchoring bias	The tendency to adhering to one’s first idea without considering other possibilities.
Confirmation bias	The tendency to tweak the information to fit one’s hypothesis.
Hassle bias	The tendency to choose a course of action that is easy or causes the least amount of stress (here, to the physician)
Rule bias	The tendency to blindly follow general rules that are arbitrarily made.
Base rate neglect	The tendency to ignore the frequency of a disease; this is especially true in the case of rare diseases.
Visceral bias	The tendency of physicians’ decisions to be influenced by feelings towards patients, which may be positive or negative.
Premature closure	The tendency of physicians to cease thinking further after making a diagnosis.
Maslow’s hammer	The tendency to over-rely on a familiar tool (e.g., endoscopy and cardiac catheterization

Ten types of cognitive bias, representative of Japan, were selected by T.W. after a careful review of the literature and a qualitative disscusion with T.H. and K.K [17–22].

### Data analysis

All statistical analyses were performed using EZR (Saitama Medical Center, Jichi Medical University, Saitama, Japan), which is a graphical user interface for R (The R Foundation for Statistical Computing, Vienna, Austria). Specifically, it is a modified version of R commander designed to add statistical functions that are frequently used in biostatistics [23]. This was used to obtain parametric statistics only; given the non-random way our data were collected and the relatively small number of cases, formal tests of differences were not attempted.

### Ethical considerations

The present study was conducted after obtaining approval from the Ethics Committee of the Shimane University School of Medicine (No. 20181017-1). All participants provided informed consent before participating in the study.

### Patient and public involvement

No patients were involved in the proceedings of this study.

## Results

**Fig. 1:**
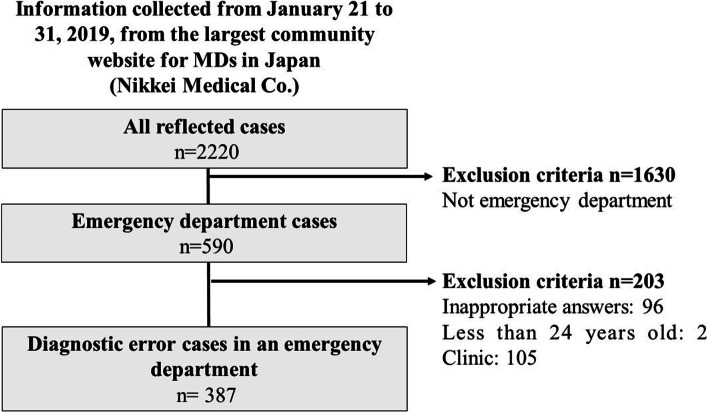
Patient diagram of cognitive bias in the emergency room for each search strategy

A total of 2220 physicians participated in the study. We excluded 1630 physicians who chose non-emergency room settings. Moreover, we excluded 96 physicians with at least one unanswered question, two physicians with incorrectly answered questions, and 105 physicians who listed their hospital size as a clinic. Ultimately, 387 physicians were included in this study (Fig. 1).

The characteristics of these 387 physicians are described in the Supplementary File. The mean age was 43 years (IQR 35-51), the mean number of years since graduation was 18 (IQR 10-26), and the mean number of years of post-graduate experience at the time of the encounter was 3 (IQR 2-5). The physician specialties were internal medicine (46.7%), surgery (14%), family medicine (6.5%), pediatrics (6.3%), and orthopedics (5.5%).

The details of the memorable diagnostic error cases in the emergency room are indicated in the Supplementary File. For the frequency of diagnostic errors, the most common hospital sizes were large and university hospitals (64.6%), the most common time of day was the night shift after 5 PM (75.9%), the most common days were Monday through Thursday (57.9%), and most common amount of time for a diagnostic error to be noticed was within a few days (79.1%).

The top five common initial diagnoses were upper gastrointestinal disease (22.7%), trauma (14.7%), cardiovascular disease (10.9%), respiratory disease (7.5%), and primary headache (6.5%). The corresponding final diagnoses for these errors were intestinal obstruction or peritonitis (27.3%), overlooked traumas (47.4%), other cardiovascular diseases (66.7%), cardiovascular disease (41.4%), and stroke (80%), respectively.

The factors that caused diagnostic errors in the emergency room were also analyzed by classifying them into environmental, information-collecting, and information-integrating factors. The most common environmental factors were work hours, physician fatigue, and work style problems, at 43.2, 27.1, and 26.6%, respectively. The most common information-gathering factors were lack of physical examination and testing, lack of history taking, and problems in interpretation of the information, at 55.6, 26.6, and 20.7%, respectively.

**Fig. 2:**
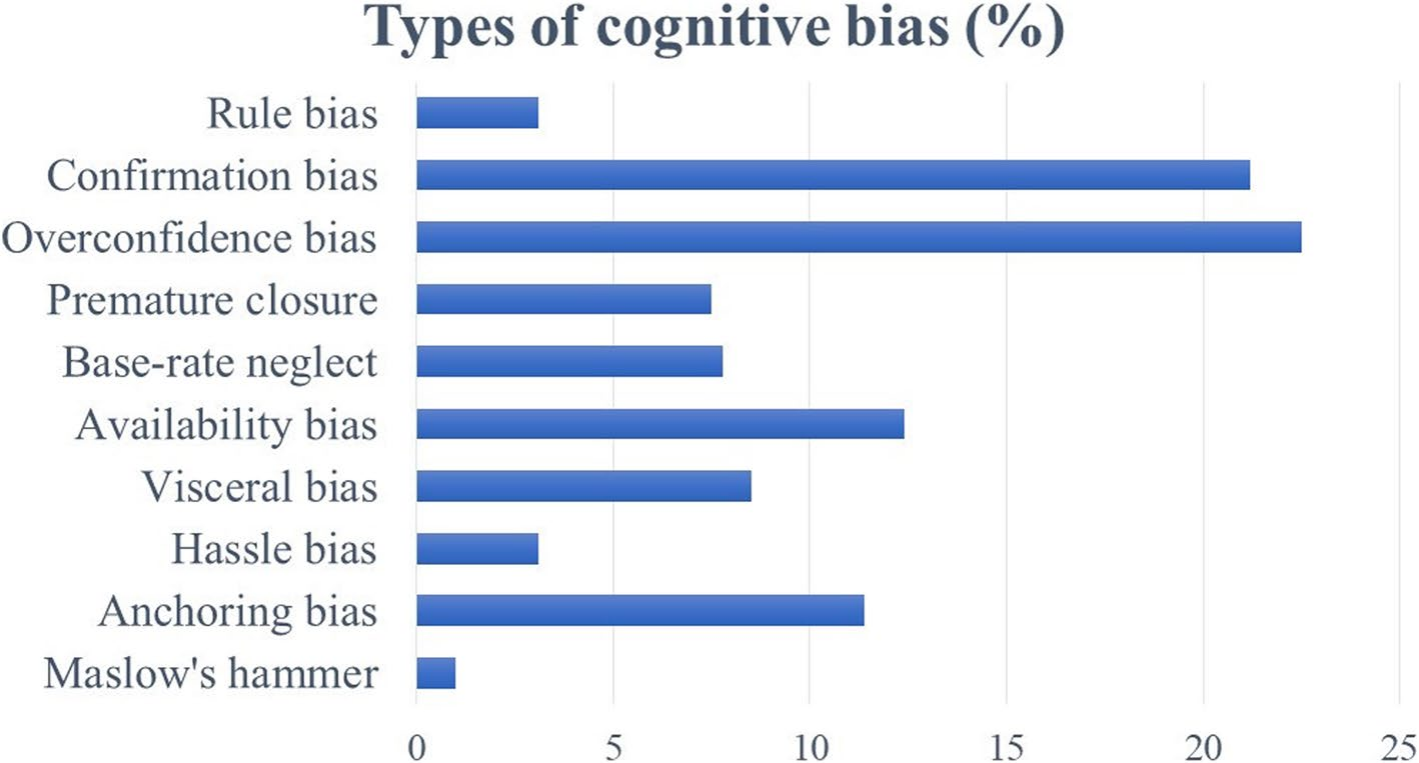
Types of cognitive bias (%)

Figure 2 shows the results of cognitive bias among the information-integrating factors. The most common type of cognitive bias was overconfidence (22.5%), making it easy to believe judgments about oneself and overconfidence. An example includes overconfidence in the decisions of the previous physician who examined the referred patient, a widespread example of bias encountered in busy clinical settings. The second most common type is confirmation bias (21.2%); this entails underestimating information that does not fit one’s hypothesis. This is followed by availability bias (12.4%): it is easy to think of things that come to mind quickly. This is also influenced by what the person has experienced recently. Anchoring bias (11.4%) refers to the idea that one becomes focused on their first thought and does not change their mind. The remainder comprises other cognitive biases (31.5%).

**Fig. 3:**
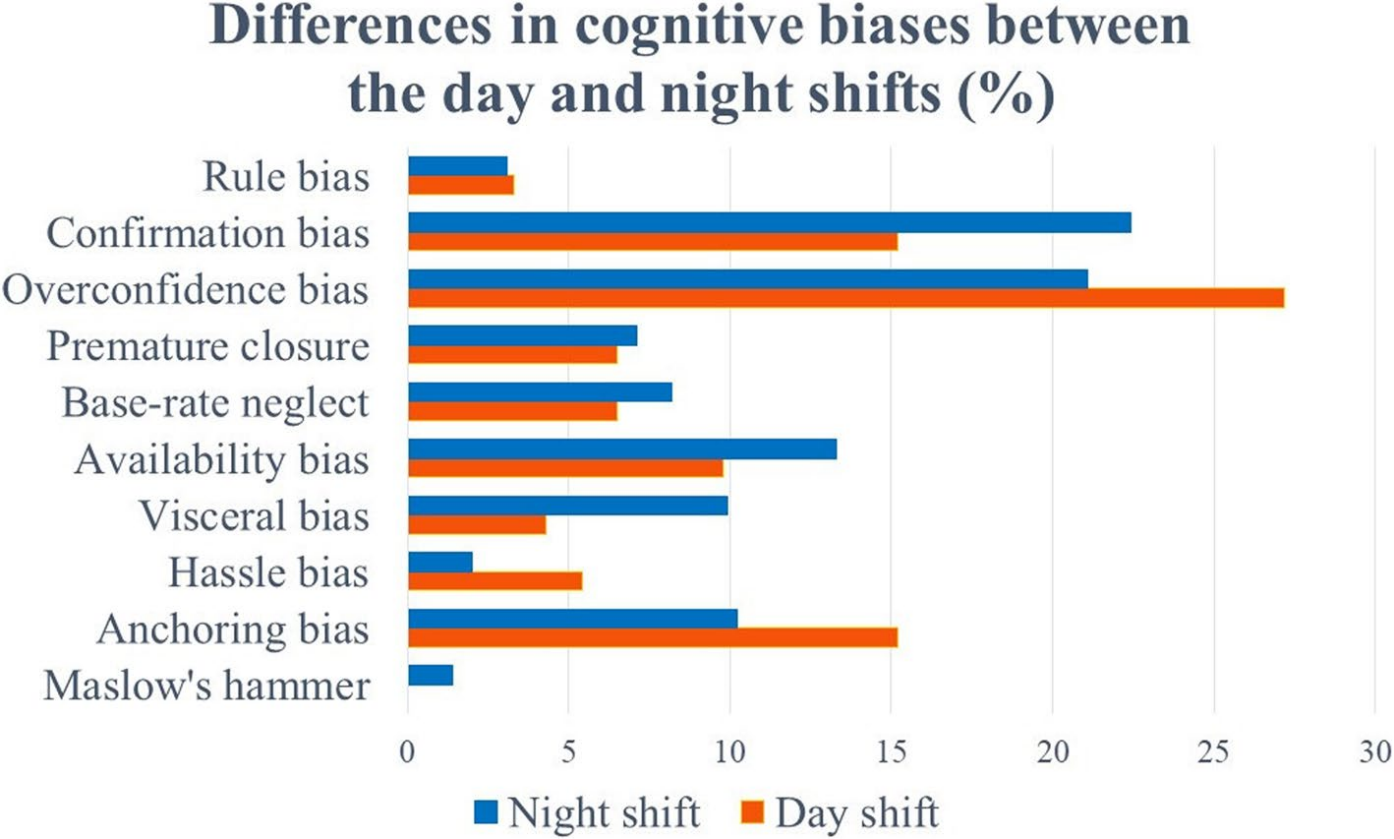
Differences in cognitive biases between the day and night shifts (%)

The differences in the types of cognitive bias between the daytime and nighttime shifts are shown in Fig. 3. The most common cognitive biases in the day (92 cases) and night shifts (294 cases) showed no significant difference (29.3 and 31.3%, respectively). Confirmation bias, premature closure, base-rate neglect, visceral bias, and Maslow’s hammer were more common at night than during the day. However, nonparametric tests showed no statistically significant differences.

**Fig. 4:**
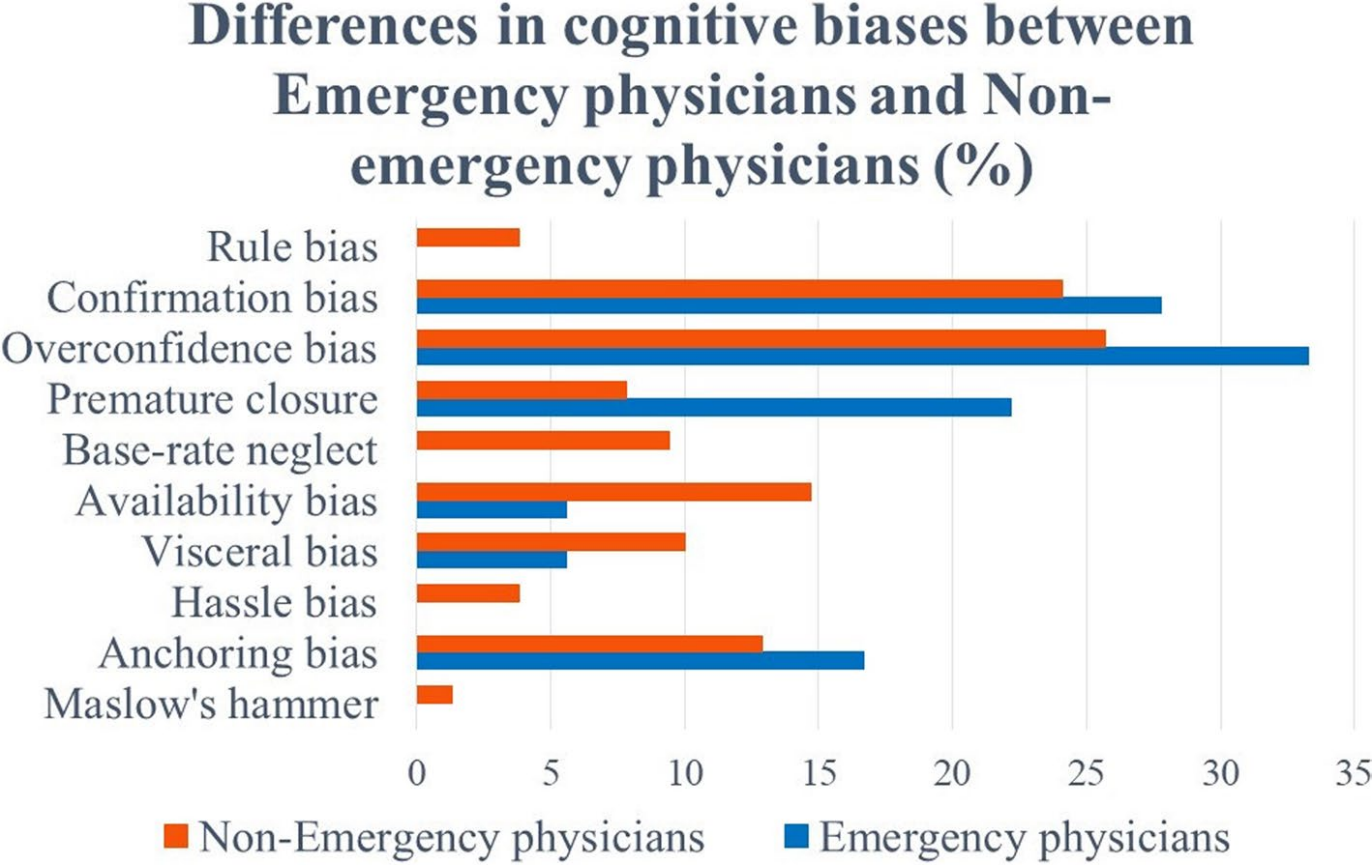
Differences in cognitive biases between emergency physicians and non-emergency physicians (%)

The differences in the types of cognitive bias between emergency and non-emergency physicians are indicated in Fig. 4. Overconfidence, confirmation, and anchoring bias as well as premature closure were more common among emergency physicians. For non-emergency physicians, rule bias, base-rate neglect, availability bias, visceral bias, hassle bias, and Maslow’s hammer were more common. However, nonparametric tests showed no statistically significant differences.

## Discussion

The most common cognitive biases involved in diagnostic errors in the emergency room were overconfidence, conformation, availability, and anchoring bias. Moreover, when initial and final diagnoses were examined, other diseases of the same organ or diseases of a nearby organ were often overlooked. To the best of our knowledge, this is the first study to quantify the types of cognitive bias that are likely to occur in emergency rooms in Japan.

In a study examining medical malpractice claims in Japan, diagnostic error-related claims (DERC) were significantly more common than non-diagnostic error-related claims (non-DERC) in the emergency room, and the mortality rate was significantly higher for DERC than for non-DERC [24]. Therefore, reducing diagnostic errors in emergency rooms is an important problem in Japan. The most common types of cognitive bias in the emergency room in previous studies were premature closure as well as anchoring, availability, and confirmation bias [1, 16, 25]. Moreover, overconfidence bias, which is overconfidence in one’s judgment, is a major factor that interferes with debiasing strategies and is reported to occur in about 15% of emergency room cases [9, 26]. The present study was consistent with the types of cognitive biases indicated in previous studies but with fewer premature closures. Premature closure is caused by stopping the thinking process after a diagnosis is made and failing to evaluate the hypothesis; it is the most common cognitive bias in diagnostic errors [27]. In this study, participants responded to the diagnostic error cases that they struggled to diagnose from their memories, which could explain lower instance of premature closure cases reported in this study. Along with these cognitive biases, aggregate bias, triage cueing, diagnosis momentum, representativeness restraint, search satisficing, psych-out error, visceral bias, posterior probability bias, gambler’s fallacy, blind-spot bias, and gender bias have also been reported as cognitive biases that are likely to occur in emergency rooms [9, 28]. However, the opposite opinion, that representativeness restraint and blind-spot bias are less likely to occur [29], has also been reported; therefore, further research is needed.

In this study, confirmation bias, premature closure, base-rate neglect, visceral bias, and Maslow’s hammer were more common during the night shift than day shift. In addition, rule bias, base-rate neglect, availability bias, visceral bias, hassle bias, and Maslow’s hammer were more common among non-emergency than emergency physicians. During the night shift, physicians are more prone to mental exhaustion due to fatigue and lack of sleep. This study also suggests that when non-emergency physicians are working in an emergency room, they may be more likely to be influenced by their own specialty or emotions from their diagnoses, or to follow incorrect rules or ignore disease frequency in an unfamiliar emergency room setting. Improving our understanding and awareness of cognitive biases is a practical first step in overcoming them [30]. A debiasing strategy is needed to overcome the cognitive biases that commonly occur in emergency rooms. Debiasing is having “adequate knowledge of alternative solutions and strategic rules for heuristic responses” and “the ability to disable System 1 processing” [31]. For premature closure, the worst-case scenario should be eliminated by asking, “What else might this be?’ [1, 28], for example, reviewing the differential diagnosis before admitting a patient or reviewing hand radiographs to look for a second fracture rather than assuming there is only one [32]. Anchoring and confirmation bias are closely related [25], and for the former, the diagnosis should be reviewed with new information and data, without preconceptions [28]. For confirmation bias, considering the opposite of the initial hypothesis [1, 28], revisiting the diagnosis if the data do not support it, and using metacognition, error theory, and cognitive coercion strategies [26] are recommended. For availability bias, it is useful to consider the objective reason for the diagnosis [28]. Other ways to reduce cognitive bias are to seek opinions outside of yourself, such as second opinions and decision support systems. A second opinion can be useful in identifying errors that might otherwise be missed and in interpreting test results. Decision support systems include checklists, flow charts, and visual aids. The availability of decision support systems and clinical information i for night shift and non-emergency physicians working the emergency room may reduce reliance on memory, and improve diagnostic reasoning performance under conditions such as stress and fatigue [32, 33].

The most common diseases that caused diagnostic errors include gastrointestinal, hepatobiliary, respiratory, cardiovascular, and infectious diseases as well as metabolic endocrinology, trauma, and malignant tumors [13, 16]. Moreover, in a study reviewing medical malpractice claims in Japan, non-hemorrhagic gastrointestinal diseases, such as gastroenteritis and intestinal obstruction were among the most common initial diagnoses in DERC cases [24]. The top four most common diagnostic error cases in this study were consistent with those of previous studies. In cases of headache, which was the fifth most common symptom, a diagnostic error for stroke was reported in 8.7% of cases [34]. Although subarachnoid hemorrhage occurs in 1–3% of patients with headaches [35], misdiagnosis or delayed diagnosis occurs in 12–51% of cases [36]. Furthermore, it has been reported that diagnostic discrepancies are associated with increased in-hospital mortality [37]. However, to the best of our knowledge, there are no studies that show the differences between the initial and final diagnostic labels; therefore, further research is needed.

## Limitations

The main limitation of this study is that the authors surveyed only the previously noted cognitive biases, which were chosen from more than 100 possible cognitive biases. Cognitive factors such as affective bias and lack of knowledge were not taken into consideration. In fact, 31.5% of the respondents answered that the reasons were not included in the indicated cognitive biases. Second, as the results were obtained from a survey of memorable diagnostic errors in the emergency room, recall bias was not eliminated. Third, it is unclear whether bias is significantly more prevalent in the emergency room because the results were not compared to other outpatient settings. Fourth, about 95% of the survey participants were non-emergency physicians; thus, we could not identify cognitive biases to which emergency physicians specifically are prone. However, the number of board-certified emergency physicians in Japan is limited [24], and non-emergency physicians need to diagnose serious diseases in the emergency room, particularly in small and medium-sized hospitals in Japan. Therefore, this study will be important in reducing medical errors in emergency rooms in Japan. Moreover, a study of educators reported that understanding the cognitive biases that are likely to occur in the emergency room [19] and learning debiasing strategies to overcome them significantly improved their ability to teach residents and improve clinical reasoning [38]. It is also useful to understand the cognitive biases that non-emergency physicians are prone to in the emergency room. Fifth, statistical comparative analysis could not be conducted in this study. This is because the study design was constructed as a descriptive study and did not involve obtaining a hypothesis-based sample size calculation; moreover, the sample size was not sufficient to perform multivariate analysis to indicate factors associated with diagnostic errors.

## Conclusions

The most common cognitive biases in the emergency room were overconfidence, confirmation, availability, and anchoring bias. Comparing the initial and final diagnoses of cases with diagnostic errors revealed that more errors were caused by overlooking another disease in the same organ or a disease in a closely related organ.

## Supplementary Information

**Additional file 1: Table S1.** Baseline Characteristics of the Survey Respondents (n=387). **Table S2.** Analysis of the most memorable diagnostic error case among physicians (n=387).

## Data Availability

The database was developed by Nikkei Medical Co.; therefore, it is not available to the public. However, upon reasonable request from the author, the data may be available with permission from Nikkei Medical Co.

## Abbreviation

DERC: Diagnostic error-related claims
