# Supplementary material for: Cognitive biases encountered by physicians in the emergency room
Source: BMC Emerg Med. 2022 Aug 26;22:148. doi: 10.1186/s12873-022-00708-3 (PMC9414136; doi:10.1186/s12873-022-00708-3)
Supplement: Supplementary file 1 — Additional file 1: Table S1. Baseline Characteristics of the Survey Respondents (n=387). Table S2. Analysis of the most memorable diagnostic error case among physicians (n=387). [file 12873_2022_708_MOESM1_ESM.docx]

Supplementary File

**Table S1. Baseline Characteristics of the Survey Respondents (n=387)**

| Age |  | 43 (IQR 35–51) |
| --- | --- | --- |
| Post-graduate years | | 18 (IQR 10–26) |
| Post-graduate year when the case was encountered | | 3 (IQR 2–5) |
| Specialties |  |  |
|  | Internal Medicine | 46.7 |
|  | Surgery | 14 |
|  | Family Practice | 6.5 |
|  | Pediatrics | 6.3 |
|  | Orthopedics | 5.5 |
|  | Neurosurgery | 4.5 |
|  | Emergency Medicine | 4.3 |
|  | Psychiatry | 2.4 |
|  | Anesthesiology | 2 |
|  | Radiology | 1.8 |
|  |  |  |

**Table S2. Analysis of the most memorable diagnostic error case among physicians (n=387)**

| Facility |  |  |
| --- | --- | --- |
|  | Small-sized hospital (20–100 beds) | 25 (6.5%) |
|  | Medium-sized hospital (101–300 beds) | 112 (28.9%) |
|  | Large/University hospital (301 or more beds) | 250 (64.6%) |
| Working hours during which the case was encountered | |  |
|  | Morning (08:30–12:00) | 28 (7.2%) |
|  | Afternoon (12:00–17:00) | 64 (16.5%) |
|  | Night shift/duty (17:00–08:30) | 294 (75.9%) |
|  | Other | 1 (0.3%) |
| Day of the week | |  |
|  | Monday–Thursday | 224 (57.9%) |
|  | Friday | 34 (8.8%) |
|  | Saturday | 37 (9.6%) |
|  | Sunday/Holiday | 92 (23.8%) |
| Time to detect the diagnostic error | |  |
|  | Within a few hours | 41 (10.6%) |
|  | Within a few days | 306 (79.1%) |
|  | Within a few weeks | 33 (8.5%) |
|  | Within a few months | 6 (1.6%) |
|  | Within a few years | 1 (0.3%) |
|  | Initial Diagnosis | Final Diagnosis |
|  | Upper gastrointestinal tract disorders (22.7%) | Intestinal obstruction, peritonitis (27.3%) |
|  |  | Cardiovascular disease (23.9%) |
|  |  |  |
|  | Trauma (14.7%) | Missed other trauma (47.4%) |
|  |  | Cardiovascular disease (19.3%) |
|  |  |  |
|  | Cardiovascular disease (10.9%) | Missed other cardiovascular disease (66.7%) |
|  |  |  |
|  | Respiratory disorders (7.5%) | Cardiovascular disease (41.4%) |
|  |  |  |
|  | Primary headache (6.5%) | Stroke (80%) |

**Questionnaire**

**Question 1.**

How often do you encounter "diagnostic errors," such as wrong diagnosis, missed diagnosis, or delayed diagnosis, including minor ones? Please choose one from the following options.

- - Almost every day
  - Several times a week
  - About once a week
  - Several times a month
  - About once a month
  - Once every few months
  - Once every six months
  - Once a year

**Question 2.**

What percentage of the time do you think you yourself encounter diagnostic errors, including minor ones? Please enter the actual number in the range of 0 to 100. (e.g., if 1 in 10 cases, enter 10% → 10)

**Question 3.**

Please recall one of the most memorable diagnostic errors in your experience (hereafter referred to as "failed cases"). What was the number of years after graduation when you encountered the case? What was your initial and final diagnosis? Please fill in the following information.

　Number of years post-graduation (please fill in the number only)

　Initial diagnosis (please provide only the name of the diagnosis)

　Final diagnosis (please provide only the name of the diagnosis)

**Question 4.**

Which of the following "3 categories of diagnostic errors" does your botched case fall under (multiple answers allowed)?

- - Miss: Missed diagnosis
  - Wrong: Different diagnosis
  - Delay: Delay in diagnosis

**Question 5.**

Please select the location where you encountered the missed case from the following options.

- - Regular outpatient clinic/first-time outpatient clinic
  - Specialty outpatient/special outpatient
  - Specialist outpatient clinic/special outpatient clinic
  - Ward
  - Treatment room / Dialysis room / Endoscopy room
  - Operating rooms
  - Procedure rooms
  - Others

**Question 6.**

Please choose one from the following options for the time of day when you encountered the diagnostic error case.

- - Morning
  - Afternoon
  - Night shift/on duty
  - Other

**Question 7.**

Please select one of the following days of the week when you encountered a failed case.

- - Monday through Thursday
  - Friday
  - Saturday
  - Holiday/Holiday

**Question 8.**

How long did it take you to reach (discover or detect) the final diagnosis of your stucco case?　Please indicate below (e.g., 30 minutes, 2 weeks, 5 years).

(e.g., 30 minutes, 2 weeks, 5 years)

**Question 9.**

In general, it is believed that three factors are involved in diagnostic errors: (1) situational (environmental) factors, (2) information gathering factors, and (3) information integration factors (cognitive bias). In the case of the mishap that you encountered, what factors do you think were involved in the situation (environment)? Please select as many that apply.

- - Physician stress
  - Time of day
  - Working style
  - Physician fatigue
  - Equipment and manpower
  - Examination system
  - Congestion
  - None of these apply to me.

**Question 10.**

What information-gathering factors do you think were involved in the failure cases? Please select as many that apply.

- - Excessive or insufficient history
  - Differences in interpretation models (ideas about causes of disease, treatment, outcome, etc.) with patients/families
  - Excessive or inadequate examination/examination
  - Differences in interpretation of useful/non-useful information
  - None of the above apply.

**Question 11.**

What information integration factors (cognitive biases) do you think were present in the failure case? Please select as many that apply. Please refer to "Typical Diagnostic Biases" (link to PDF) for an explanation of each cognitive bias.

- - Availability bias
  - Overconfidence bias
  - Anchoring bias
  - Assurance bias
  - Hustle bias
  - Rule bias
  - Ignorance of frequency
  - Negative/positive (instinctive bias)
  - Early closure
  - Maslow's hammer
  - None of the above apply.

**Question 12.**

What is the ratio of the factors you answered in questions 9 to 11?

Ratio of information gathering factors (fill in whole numbers so that the total equals 10)

Percentage of information integration factors (cognitive biases) (fill in whole numbers so that the total sums to 10)

**Question 13.**

Please select from the following options the primary facility to which the doctor belongs.

- - Non-floor clinic
  - A clinic with beds (1-19)
  - Small hospital (20-99 beds)
  - Medium-sized hospital (100-299 beds)
  - Large hospital (300 or more beds)
  - Others

**Question 14**

What is your primary specialty?

- General Medicine
- General Internal Medicine
- Gastroenterology
- Cardiovascular Medicine
- Respiratory Organs Internal Medicine
- Neurology
- Metabolism and Endocrinology
- Diabetology
- Nephrology
- Hematology
- Allergy
- Rheumatology
- Infectious Diseases
- Internal Medicine Departments (other than the above)
- General Surgery
- Gastroenterological Surgery
- Cardiovascular Surgery
- Respiratory Surgery
- Pediatric Surgery
- Neurosurgery
- Orthopedic Surgery
- Plastic Surgery
- Surgical Departments (other than the above)
- Dermatology
- Urology
- Obstetrics & Gynecology
- Pediatrics
- Ophthalmology
- Otorhinolaryngology
- Emergency Medicine
- Psychiatry
- Radiology
- Anesthesiology
- Rehabilitation
- Pathology
- Laboratory Medicine
- Other Departments
- Junior Resident

**Question 15.**

Please select the age of the doctor.

- 20s
- 30s
- 40s
- 50's
- 60s
- 70's
- 80 years old and over

**Question 16.**

How many years has it been since you graduated from medical school? Please enter the number of years since graduation, numbers only.
